# Schools’ We-mentality and Students’ Civic Engagement – A Text-based Approach

**DOI:** 10.1007/s12187-022-09954-0

**Published:** 2022-08-31

**Authors:** Hendrik Hüning

**Affiliations:** Department of Economics, Hamburg University, Von-Melle-Park 5, 20146 Hamburg, Germany

**Keywords:** Civic engagement, Sense of community, School climate, Text as data

## Abstract

This paper studies the role of schools' we-mentality in shaping students' civic outcome. A school's we-mentality is important for the students' perception and education of sense of community. We-mentality is measured by an automated content-analysis approach applied to the schools' general principle. Data stem from a survey conducted in 13 German schools with 488 students. Using OLS and multi-level regression techniques, I find that stronger we-mentality is associated with more students being engaged in local civic activities. Moreover, students that exhibit stronger trust in others and are willing to engage with new and unknown tasks show more positive attitudes towards civic issues. The results hold relevance for the educational design of schools in fostering adolescents' civic education and participation.

## Introduction

Civic participation is a cornerstone of democratic societies. The interest, attitudes and engagement of individuals towards civic issues fundamentally shape the functioning of our democratic systems (Putnam, 2000). Thus, it is not surprising that scholars from various disciplines are interested in the contextual factors and conditions that shape attitudes and engagements towards civic issues (Arvanitidis, 2017).

Schools are considered to play an important role as institutions educating young people on democratic principles and to serve as niches for the development of civic engagement (Guillaume et al., 2015). Understanding the influence of different actors, activities and interactions that shape political participation and engagement of the youth is crucial for developing standards of civic education (Dudley & Gitelson, 2002).

This paper studies the effect of individual- and school-level characteristics on civic attitudes and engagement of 488 students from 13 German schools. Students’ civic attitudes and engagement are measured through eight survey questions assessing their attitudes towards their own role in society, their engagement at and outside of school with regard to honorary offices and their local and online engagement with regard to political or societal issues. School-level ‘we-mentality’, as a school’s intention and educational approach to promote we-ness at school, is captured performing a dictionary-based content analysis on the schools’ general principles (German: Leitbild) that are published on the schools’ corresponding homepages. This approach contributes to the growing economic and political science literature using “text as data” (Gentzkow et al., 2019).

The paper contributes to the literature on the role of individual characteristics and schools’ contextual factors on students’ civic behavior. With regard to individual characteristics, Castillo et al. (2015) showed that children from families with lower socioeconomic status exhibit less political participation. Students’ civic engagement is not only affected by their family background but also by their interaction with peers. Luengo Kanacri et al. (2017) demonstrated that students’ pro-social behavior towards close peers constitute foundations for later civic engagement. Moreover, civic engagement has been shown to be more pronounced for students with stronger sense of belonging to the school and its community (Encina & Berger, 2021).

With regard to contextual factors, recent studies have found a positive effect of a school’s social climate on students’ civic behavior. Castillo et al. (2015) investigated the role of civic knowledge and classroom climate on political participation and found a positive influence. Jagers et al. (2017) studied the role of classroom climate on civic engagement of Black and Latino middle school students. They found that equitable school climate predicts higher civic attitudes one year later. Moreover, research suggests that a school’s climate cannot only directly affect students’ civic outcomes but also as a moderating factor. For instance, in an empirical study with students from middle schools, Guillaume et al. (2015) found that individual positive perceptions of a school’s climate are positively related to school connectedness that in turn affects civic engagement. Schulz et al. (2017) found a positive association between classroom climate, which they measured as students’ perception of the openness of classroom discussions about political and societal issues, and students’ interest in political and societal issues. More recently, Encina and Berger (2021) found that a school’s social climate can effectively moderate students’ sense of belonging and valuing of the school that in turn fosters their civic behavior. Understanding these interactions between a school’s climate, individual characteristics and their civic engagement is of major interest to develop measures of civic education.

As previous research suggests, a school’s social climate is not clearly defined and empirical studies came up with conceptual approaches that focus on different components and processes (Encina & Berger, 2021). For instance, while Guillaume et al. (2015) and Quin (2017) used students’ individual perceptions of teacher-student or student-student relationships, Jagers et al. (2017) used students’ perceptions of equitable treatment of racial, socioeconomic, and gender groups. In contrast, Encina and Berger (2021) measured school climate on the teacher- and school-staff level by asking about the schools’ disciplinary structure and student support. Their measure rests on the theory of authoritative school climate.

In contrast to previous studies, this study investigates a school’s we-mentality as an important ingredient for a school’s social climate. I define we-mentality as a school’s intention and willingness to promote a feeling of we-ness or togetherness among teachers and students as one of their guiding principles. Empirically, we-mentality is measured by applying automated content analysis, i.e. a dictionary approach that detects we-mentality in natural language text, to the schools’ general principle (German: Leitbild) that is published on the schools’ homepages. A general principle is a school’s self-description that summarizes the pedagogical goals, teaching convictions and focuses. We-mentality that is expressed in the general principle captures part of a school’s “implemented curriculum” (Akker 2004; Bron & Thijs, 2011). The curriculum perspective of education distinguishes (a) the *intended curriculum* that is predetermined by education authorities (b) the *implemented curriculum*, that is actually teached at schools and (c) the *attained curriculum* that is actually achieved by students. Thus, the study investigates how the implemented curriculum, i.e. how important a school deems we-mentality in their educational approach, relates to the attained curriculum, i.e. students’ civic attitudes and engagement.

Finally, from a theoretical perspective, this work relates to the psychological concept of sense of community (SOC). While McMillan and Chavis (1986) formulated a four-dimensional framework with membership, influence, needs fulfillment, and shared emotional connection as the driving forces of sense of community, Nowell and Boyd (2010, 2014) introduced human needs theory to the concept of SOC and distinguished community as a resource and responsibility.

Building on the concept of sense of community as a responsibility, Procentese et al. (2019) developed the concept of responsible togetherness (SoRT). The authors define SoRT as “structural opportunities within the community context, shared norms and individual perceptions of the community, which are all aspects underlying the idea of SoRT, can determine whether individuals will take responsibilities in thinking and enacting changes within their community” (page 258). In a survey among university students, the authors found that SoRT has an indirect effect on student’s civic participation via their sense of community. Relatedly, Prati et al. (2020) investigate sense of community responsibility (SOC-R). Using explanatory and confirmatory factor analysis, the authors found that SOC and SOC-R are “two separate, albeit related, constructs” (page 1). Their results support the model where community experience is a function of resource and responsibility components.

Beyond the literature on SOC and its components and determinants, also other research describes the importance of “we-ness” and “togetherness” for the concept of belonging. For instance, Klückmann (2016) describes a concept of community as a “group of people sharing the feeling of we-ness” (page 32). Kagan et al. (2007) state that community “exists through shared meaning” (page 75). Thus, togetherness is seen to be an important factor for forming a community and sense of belonging.

Importantly, as Procentese et al. (2019) point out: The direction of the effects between SOC, SoRT and participation is still unclear (page 249). Does sense of community or SoRT promote participation or vice versa? By measuring we-mentality from schools’ general principles, the current study avoids identification problems. While we-mentality formulated in the schools’ teaching approach and convictions can be theorized to affect students’ civic attitudes and engagement (assuming that those statements from the general principle are at least partly put into practice), it is implausible that students’ civic attitudes and engagement affect “we-words” that are used in the general principle. In other words, the measure that I construct from the general principles is exogenous and allows investigating one of the two potential effect directions.

Sense of community concepts have been investigated in different environments such as workplace (Brodsky & Marx, 2001), religious communities (Miers & Fisher, 2002) and student communities (Pretty, 1990). The school as a community is special in this context because sense of community cannot only be experienced in schools but the schools’ educational approach might explicitly teach the value of community as a resource and responsibility through their activities and value of togetherness. As Nowell and Boyd (2014) pointed out: “there is still much left under-theorized and untested about the experience of community and the mechanisms through which these perceptions and experiences translate into action” (p. 239). This paper highlights the channel of we-mentality (promoting togetherness) through which sense of community at schools can be experienced and educated.

The remainder of the paper is structured as follows: Section 2 introduces the survey design and empirical strategy. Section 3 presents the data. The empirical results are summarized in Section 4 and discussed in Section 5. Section 6 concludes.

## Methods

### Survey Design

For the survey, students from secondary schools in Berlin and Hamburg were recruited. Local school ministries in Berlin and Hamburg gave approval for conducting the survey among the senior years of the secondary school (German: Gymnasiale Oberstufe). Therefore, students were from 11th or 12th grade and were at least fifteen years old. Overall, 214 schools have been contacted by phone and afterwards informed about the survey and its procedures in written form by E-mail. Sixteen schools agreed to participate. The survey was conducted between December 2019 and March 2020 in thirteen of these schools. Four of those are located in Berlin and nine are located in Hamburg. Unfortunately, in March 2020, the fieldwork had to be stopped because of the Covid-19 pandemic and school closings. For this reason, the survey could not be conducted in the remaining three schools.

The survey was conducted in schools during the students’ regular lessons. The survey was entirely computer-based, i.e. students separately used a computer or laptop to participate in the survey. The computer infrastructure was either provided by the schools themselves or tablets were provided by the researchers via the mobile laboratory of the WISO-lab at Hamburg University. The program for the survey was designed using the software o-tree (Chen et al., 2016). Parents and students were informed about the procedures of the study two weeks in advance. Written consent was obtained from all students before the study took place. In case of underage students, parents or legal representatives had to give written consent, too. The survey was not incentivised.

### Regression Analysis

With regard to the empirical strategy, I use Ordinary Least Squares regressions (OLS) and multi-level regressions to investigate the effects of individual- and school-level characteristics on students’ civic attitudes and engagement. OLS regressions are run with and without school fixed effects in order to investigate how much of the variation of students’ civic attitudes and engagement can be explained by the school. Subsequently, the effects of school-level characteristics is analyzed using multi-level regressions. More specifically, the effect of we-mentality, the variable of interest, is investigated in three benchmark regressions on three outcome variables. Inference on these benchmark regressions is corrected for multiple hypothesis testing (MHT) using the conservative Holm-method (Holm, 1979). I perform several robustness checks with regard to these benchmark regressions. All regressions were performed using the software tool R.

## Data

### Student-Level Data

Overall, 501 students in 19 sessions participated in the survey. Due to technical malfunction, data from 13 students had to be dismissed, leaving 488 observations for the analysis. Table 1 presents some descriptive characteristics and Table 2 provides the age distribution of the sample. Students were between 15 and 21 years old, averaging 17 years. Most of the students were between 16 or 17 years old (76.6%) as it is typical for 11th and 12th graders. Overall, 56% of the students are female and one student is diverse. From all participating students, 27% went to schools in Berlin. Almost all students are born in Germany (96%).

**Table 1:** Summary statistics

Variable	Mean	St. Dev	Min	Max
Age (in years)	17	0.91	15	21
Female	56	50	0	100
School in Berlin	27	44	0	100
Born in Germany	96	20	0	100
Pocket money (in Euro)	24.89	39.36	0	450
School size (no. of students)	805	239	159	1075
Private school	31	46	0	100
Catholic school	23	42	0	100
Music school	19	39	0	100
Bilingual school	25	43	0	100
Natural science	17	37	0	100

The number of observations is 488. Numbers are in percent except otherwise indicated. The variables Female, School in Berlin, Born in Germany, Private school, Catholic school, Music school, Bilingual school and Nautral science are dummy variables

**Table 2:** Age distribution

Age	Frequency	Percentage
15	3	0.6
16	143	29.3
17	231	47.3
18	82	16.8
19	22	4.5
20	5	1.0
21	2	0.4

The table displays frequencies and percentages per years of age

Moreover, eight survey items were developed that capture students’ civic attitudes and engagement. The survey items were developed by the author and tested in a pilot study with first semester students at the university in advance of the survey in schools. Following the reasoning in Kahne and Sporte (2008), these items reflect community-based forms of civic attitudes and engagement rather than more formal forms of political activities such as working on campaigns, engagement within parties or voting. Young students less likely engage in formal political action making a broader perspective of civic engagement necessary. The items reflect their general attitudes towards society and their engagement with a local and online community with regard to societal issues. The items are summarized as follows (See Appendix Table 7 for the exact reading of the items):

- the importance of giving something back in society (Variable name: *soc_return*)
- the importance of being informed about what is happening in the society (Variable name: *informed_soc*)
- the students’ own role in making a change in society (Variable name: *change_soc*)
- being a member in a club or association (Variable name: *mem_club*)
- having taken a school office such as elected representative of the pupils (Variable name: *school_off*)
- having taken honorary post outside of school matters (Variable name: *hon_out_school*)
- writing letters to magazines/newspapers or writing a comment online on a (news)page with regard to societal or political topics (Variable name: *write_let*)
- having online discussions with others on social media regarding political or societal issues (Variable name: *onl_media*)

These eight items reflect a student’s individual attitudes and engagement with regard to civic issues. The last two items, i.e. *write*_*let* and *onl_media*, account for “distance” and online participation in political and civic discourse and engagement. In modern societies and the age of the internet, political participation more often means taking part in online discussions or organizing political protest online.^1^ As Nelson et al. (2017) showed for the United States, digital civic engagement nowadays often substitutes more conventional (i.e. local) civic engagement.

In order to reduce the dimensionality of these individual measures, principal component analysis (PCA) is used to reduce the eight measures to a lower number of factors, i.e. components, that capture most of the variance of the original items. First, I investigate if the eight measures are suitable for a PCA. The Kaiser–Meyer–Olkin (KMO) criterion for these eight measures takes the value 0.67 indicating substantial correlations between the measures to justify the use of PCA. Second, investigating the scree plot (see Appendix Fig. 2) indicates that three components are sufficient to represent the eight measures, i.e. three eigenvalues are above one.

Results of a PCA with three components are depicted in Appendix Table 9. The variables *informed_soc*, *change_soc* and *soc_return* display strong loadings on component one. The variables *mem_club*, *hon_out_school* and *school_off* have strong loadings on component two and finally, the variables *write_let* and *onl_media* have strong loadings on component three. Thus, component one generally reflects students’ attitudes towards civic issues. In the following, component one is called *attitudes*. Component two seems to reflect students’ engagement in and outside of schools, i.e. in their local environment and is denoted as *eng_local*. Finally, component three reflects students’ engagement with newspapers and online comments. Component three is denoted as *eng_onl*. Subsequently, *attitudes*, *eng_local* and *eng_onl* are used as outcome variables in OLS and multi-level regressions to investigate the effect of school characteristics on civic attitudes and engagement.

In order to explain students’ civic attitudes and engagement, the following student-individual attitudes and characteristics were collected through the survey (compare Appendix Table 8). A student’s perception of the social status of the parents (*social*_*ladder_parents*), individual attitudes towards refugees (*refugee_attitudes*), their willingness to spend some time abroad after school (*prob_abroad*), willingness to donate to a charity (*donation*), trust in others (*trust_others*), willingness to engage with tasks that might not be solvable (*solvable_tasks*), and finally attitudes towards party-democracy and the European Union (*partydemo*_*attitudes*, *eu_attitudes*) as well as students’ willingness to further engage with issues related to party-democracy and the European Union (*partydemo_willingness* and *eu_willingness*). Finally, *age* is numeric and controls for students’ age and *female* is a dummy variable that is equal to one for female students and zero otherwise. These variables are used as individual-level predictors in the regressions.

### School-Level Data

With regard to school characteristics, the following publicly available data are collected and used to construct the following variables. First, the variable *private* is equal to one if a school is funded by a private institution and zero otherwise. Second, the variable *catholic* is equal to one for schools that have catholic principles and zero otherwise.^2^ Third, *music* is equal to one for schools that have a strong focus on music, i.e. students dedicate a substantial time at school learning an instrument, and zero otherwise. Fourth, *naturalscience* is equal to one for schools that have a focus on natural sciences, i.e. the school promotes a strong education in mathematics, physics, chemistry and biology, and zero otherwise. Fifth, *bilingual* is equal to one for schools that offer bilingual education, i.e. some of the classes the students have to attend are teached in English or another European language, and zero otherwise. Sixth, the variable *size* controls for the size of the schools, i.e. the number of students.

Table 1 summarizes these characteristics. On average, a school in our sample has 805 students. With regard to the organizational structure and funding, 31% of students attend a private school, 23% attend a catholic school. Moreover, 19% attend a school with strong focus on music, 17% with a focus on natural sciences and 25% a school that teaches some classes in a foreign language (bilingual schools).

Beside these indicator and quantitative variables, a qualitative measure from schools’ general principles (German: Leitbild) is extracted. The general principle is publicly available on each school’s website. It states a school’s educational goals, general teaching ideas and convictions, ethos and focus of teaching, if applicable. From these textual data, I extract we-mentality with a dictionary approach.^3^ More specifically, all words in a given general principle are counted that are associated with togetherness such as the words “collaboration”, “helpfulness” or “mutual”. The hypothesis that I want to test is: The more a school is governed by we-mentality, the more positive students’ attitudes are towards civic issues and the more students are civically engaged.

The variable *we-mentality* is defined as the share of words in school i’s general principle that are associated with a “we-together-culture”, i.e. the number of togetherness words divided by the total number of words in that same general principle (See the full word list of *we-mentality* in Appendix Table 10). More formally,

1 $${We\text{-}mentality}_{i}=100*\frac{{WeWords}_{i}}{{TotalWords}_{i}.}$$

Thus, we-mentality is the percentage of words that can be attributed to “togetherness”. Needless to say that using this measure assumes that a school’s ethos and community principles that are put into practice by school officials and teachers are (at least partly) expressed in its general principle. Compared to asking teachers and students directly about their feeling of togetherness in class or at school, this measure is less prone to social desirability bias. Although a school’s general principle might also be prone to social desirability, it is less obvious how this relates to the use of “we-words” in it.

Table 3 provides an overview over the number of we-words and total words as well as the measure of we-mentality for each of the 13 schools of the sample. We-mentality ranges from 0.17% to 0.89%. Although the absolute values are quite low, the differences across schools are quite remarkable.

**Table 3:** Overview over we-mentality in schools’ general principle

School id	We-words	Total words	We-mentality (in %)
1	3	1716	0.17
2	6	3525	0.17
3	29	5899	0.49
4	67	7529	0.89
5	20	2261	0.88
6	21	4297	0.49
7	46	8625	0.53
8	18	5569	0.32
9	67	10,589	0.63
10	20	3510	0.57
11	13	3201	0.41
12	15	5318	0.28
13	6	981	0.61

The table displays frequencies and percentages of we-words per school

**Fig. 1:**
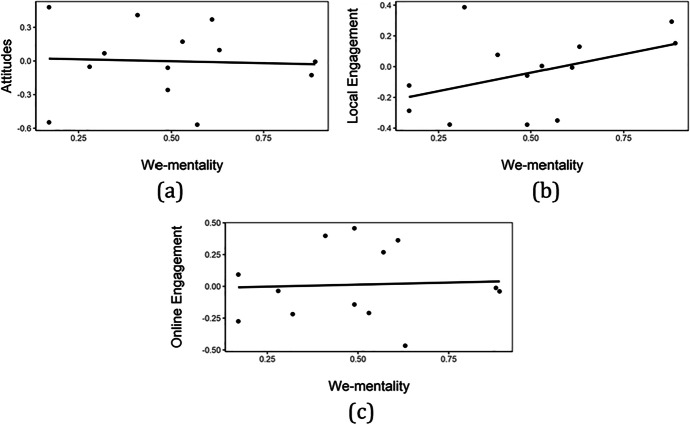
Association between we-mentality and civic attitudes and engagement. Horizontal axes display we-mentality (in %) as displayed in Table 2. Vertical axes display the three principal components from the principal component analysis (PCA) that are non-dimensional. (**a**) Attitudes (**b**) Local engagement (**c**) Online engagement

Before turning to regression analysis, the association between a school’s we-mentality and students’ average civic attitudes and engagement is illustrated. Results are depicted in Fig. 1. Panel b) shows that there is a strong positive association between a school’s we-mentality and the average local engagement of students. The association between we-mentality and attitudes and online engagement are a lot less strongly pronounced (panel a) and c)). This is confirmed by Pearson’s correlation coefficients that are -0.05, 0.44 and 0.05, respectively.

The variables *catholic*, *private*, *music*, *naturalscience*, *bilingual*, *size* and the main variable of interest, *we-mentality*, are subsequently used as school-level predictors in regressions to investigate their association with students’ civic attitudes and engagement.

## Results

First, I investigate the effect of student-level characteristics on civic attitudes and engagement. The principal components *attitudes* (*informed_soc*, *change_soc* and *soc_return*), *eng_local* (*mem_club*, *hon_out_school* and *school_off*) and *eng_onl* (*write_let* and *onl_media*) serve as the dependent variables. Results are depicted in Table 4.

**Table 4:** Civic attitudes and engagement – OLS

	attitudes	attitudes	eng local		eng_local	eng_onl	eng_onl
female	0.140	0.132	− 0.084		− 0.037	− 0.105	− 0.159
	(0.089)	(0.095)	(0.096)		(0.101)	(0.094)	(0.098)
age	− 0.060	− 0.016	0.006		0.100*	0.085*	0.014
	(0.047)	(0.055)	(0.053)		(0.057)	(0.051)	(0.057)
social ladder parents	− 0.034	− 0.047	0.092		0.084	− 0.041	0.017
	(0.055)	(0.057)	(0.065)		(0.066)	(0.063)	(0.063)
refugee attitudes	0.060^∗∗^	0.041	− 0.049^∗^		− 0.046	0.061^∗∗^	0.061^∗∗^
	(0.027)	(0.027)	(0.029)		(0.030)	(0.029)	(0.029)
prob abroad	0.033	0.028	0.119^∗∗∗^		0.090^∗∗^	0.048	0.074^∗^
	(0.037)	(0.037)	(0.038)		(0.038)	(0.038)	(0.038)
donation	0.008	0.003	0.017		0.014	0.003	0.005
	(0.011)	(0.011)	(0.012)		(0.011)	(0.012)	(0.012)
trust others	0.122^∗∗∗^	0.120^∗∗∗^	0.029		0.043	− 0.043	− 0.001
	(0.039)	(0.040)	(0.042)		(0.043)	(0.044)	(0.042)
try new things	0.127^∗∗^	0.118^∗∗^	0.031		0.002	0.047	0.066
	(0.052)	(0.052)	(0.054)		(0.054)	(0.055)	(0.054)
solvable_tasks	0.009	0.018	0.082^∗^		0.121^∗∗∗^	0.011	0.006
	(0.040)	(0.041)	(0.046)		(0.045)	(0.046)	(0.044)
partydemo attitudes	0.075^∗∗∗^	0.067^∗∗∗^	0.021		0.019	− 0.038	− 0.028
	(0.024)	(0.024)	(0.028)		(0.028)	(0.027)	(0.027)
eu attitudes	0.022	0.022	0.012		0.022	− 0.015	− 0.004
	(0.026)	(0.026)	(0.029)		(0.028)	(0.030)	(0.030)
partydemo willingness	0.212^∗∗∗^	0.198^∗∗∗^	0.052		0.058	0.161^∗∗∗^	0.168^∗∗∗^
	(0.042)	(0.042)	(0.052)		(0.051)	(0.051)	(0.050)
eu willingness	0.102^∗∗^	0.104^∗∗^	0.030		0.023	0.004	− 0.012
	(0.052)	(0.052)	(0.052)		(0.050)	(0.054)	(0.051)
constant	− 2.360^∗∗∗^	− 2.572^∗∗^		− 1.450	− 3.455^∗∗∗^	− 2.240^∗∗^	− 1.901^∗^
	(0.884)	(1.032)		(0.976)	(1.039)	(0.931)	(1.030)
Obs	485	485		485	485	485	485
R2	0.287	0.311		0.066	0.117	0.064	0.148
School FE	No	Yes		No	Yes	No	Yes
F Statistic	14.613^∗∗∗^	8.268^∗∗∗^		2.564^∗∗∗^	2.426^∗∗∗^	2.493^∗∗∗^	3.178^∗∗∗^

The table reports results of Ordinary Least Squares regressions (OLS) with *attitudes*, *eng_local* and *eng_onl* as the dependent variables. Regressions are once shown with and without school fixed effects (School FE) included. Regression coefficients are presented together with heteroscedasticity-consistent standard errors reported in parentheses. * indicates significance at the 10% level, ** at the 5% level and *** at the 1% level

The results demonstrate that students’ individual trust in others exhibits a positive and significant effect on attitudes towards civic issues. Students that are willing to engage with new and unknown matters (variable *try_new_ things*) exhibit more positive attitudes towards civic issues. The findings of trust in others and willingness to try new things being positively associated with attitudes towards civic issues is intriguing. The mechanisms being at work here, however, remain unclear from the methodological approach at hand. A potential explanation could be that students that exhibit strong trust in others or that are willing to try new things are more self-confident, show a higher level of maturity or are more integrated at school or in their community. Finally, positive attitudes towards party-democracy and willingness to further engage with this topic, is positively related to civic attitudes. The latter is also related to students’ online civic engagement.^4^

Moreover, as the *R*^2^ indicates, adding school fixed effects to the OLS regressions substantially improves the model fit (See column 2,4 and 6). This is a first indication that the school environment is also related to individual attitudes and engagement towards civic issues.

In the standard multivariate OLS framework, however, it is not possible to estimate the effects of observed and unobserved school characteristics separately. This framework assumes each observation, i.e. a student, to be independent. If the school has an influence on students’ civic attitudes and engagement, it is, however, reasonable to assume that students from the same school are more similar with regard to civic attitudes and engagement than students from different schools. In the following, multi-level regressions are applied that specifically account for the fact that students are “clustered” within a school that also might exhibit an influence on their civic attitudes and engagement. The school is considered to be the second level of a two-level model.^5^

Before investigating the effect of school characteristics on individual attitudes and engagement towards civic issues, I test if there is substantial variation across schools with regard to our outcome variables to justify the use of a multi-level approach. For this, the random intercept model and the intraclass correlation coefficient (ICC) are reported. Results are depicted in Table 5. The ICC indicates that between 3 and 8% of the variability in individual attitudes and engagement towards civic issues can be attributed to differences in schools. Thus, a substantial share of variation can be explained by the school. This serves as a first justification for the use of a multi-level approach, where the distinct clustering of students within schools is accounted for. A test for the significance of the random effects in the three models in Table 5 further supports the use of a multi-level approach (p-values are 0.000, 0.029 and 0.000, respectively).

**Table 5:** Civic attitudes and engagement (Multi-level with random intercept only)

	attitudes	eng_local	eng_onl
Constant	0.004	− 0.019	0.015
	(0.091)	(0.066)	(0.080)
Obs	487	487	487
Groups (school id)	13	13	13
ICC	0.08	0.03	0.05
AIC	1361.60	1382.77	1372.50
BIC	1374.16	1395.34	1385.07
Log Likelihood	-677.80	-688.39	-683.25

The table reports results of multi-level regressions (with random intercept only) with *attitudes*, *eng_local* and *eng_onl* as the dependent variables. Regression coefficients are presented together with standard errors reported in parentheses. * indicates significance at the 10% level, ** at the 5% level and *** at the 1% level

Since a substantial proportion of the variation in students’ civic attitudes and engagement is explained by the school, I investigate the effect of specific school characteristics in more detail by adding explanatory variables on both levels to the random intercept model. Results are depicted in Table 6. As the table shows, the previous findings with regard to the effects of individual characteristics, i.e. the effect of trust in others, the willingness to try new things and attitudes and willingness to engage with party-democracy, are robust to the model choice and remain highly significant within the multi-level framework.

**Table 6:** Multi-level with individual- and school-level predictors

	attitudes	eng_local	eng_onl
female	0.111	− 0.044	− 0.148
	(0.085)	(0.096)	(0.094)
age	− 0.044	0.094^∗^	− 0.004
	(0.048)	(0.054)	(0.053)
social ladder parents	− 0.040	0.095	0.010
	(0.058)	(0.065)	(0.064)
refugee attitudes	0.049^∗^	− 0.047^∗^	0.060^∗∗^
	(0.026)	(0.029)	(0.028)
prob abroad	0.034	0.093^∗∗^	0.064^∗^
	(0.033)	(0.037)	(0.036)
donation	0.007	0.013	0.005
	(0.010)	(0.012)	(0.011)
trust others	0.114^∗∗∗^	0.035	− 0.001
	(0.039)	(0.044)	(0.043)
try new things	0.131^∗∗∗^	0.010	0.059
	(0.046)	(0.052)	(0.051)
solvable tasks	0.015	0.117^∗∗∗^	0.008
	(0.040)	(0.045)	(0.044)
partydemo attitudes	0.068^∗∗∗^	0.020	− 0.032
	(0.025)	(0.027)	(0.027)
eu attitudes	0.023	0.021	− 0.006
	(0.027)	(0.030)	(0.029)
partydemo willingness	0.204^∗∗∗^	0.059	0.168^∗∗∗^
	(0.044)	(0.050)	(0.049)
eu willingness	0.105^∗∗^	0.018	− 0.007
	(0.047)	(0.053)	(0.052)
size	− 0.0001	0.001^∗∗∗^	− 0.001^∗∗∗^
	(0.0002)	(0.0002)	(0.0002)
we-mentality	0.189	0.839^∗∗∗^	0.199
	(0.282)	(0.316)	(0.310)
private	− 0.265^∗∗^	0.182	− 0.300^∗∗^
	(0.134)	(0.150)	(0.147)
catholic	0.086	0.276	0.458^∗∗∗^
	(0.161)	(0.180)	(0.176)
music	0.015	0.337	− 0.590^∗∗∗^
	(0.187)	(0.209)	(0.205)
naturalscience	− 0.041	− 0.274	0.177
	(0.161)	(0.180)	(0.177)
bilingual	0.031	0.497^∗∗∗^	0.057
	(0.147)	(0.165)	(0.162)
constant	− 2.380^∗∗^	− 4.276^∗∗∗^	− 0.346
	(0.978)	(1.096)	(1.075)
Obs	485	485	485
Groups (school id)	13	13	13
ICC	0.00	0.00	0.00
Log Likelihood	− 604.152	− 659.099	− 649.839
AIC	1,254.304	1,364.197	1,345.678
BIC	1,350.539	1,460.433	1,441.914

The table reports results of multi-level regressions with *attitudes*, *eng*_*local* and *eng_onl* as the dependent variables. Explanatory variables are on the individual- and school-level. ICC (intraclass correlations) corresponds to the variation between schools that remain unexplained by the model. Regression coefficients are presented together with standard errors reported in parentheses. * indicates significance at the 10% level, ** at the 5% level and *** at the 1% level

With regard to school-level characteristics, I find that schools that are funded by the catholic church are associated with students that are more engaged with online civic issues. Students who attend schools that offer bilingual education are associated with more local engagement. Students from private schools relate to less positive attitudes with regard to civic issues than those from public schools. Moreover, students from private schools are associated with significantly less engagement online than those from public schools. Local engagement within or outside of school, however, is positively but insignificantly related to *private*. This last finding contrasts with that of Encina and Berger (2021) who found a positive and significant effect of private school administration on students’ civic behavior within schools.

Finally, with regard to we-mentality, the main variable of interest, I find a positive association with students’ local civic engagement. I do not find, however, that we-mentality affects students’ attitudes towards civic issues or online engagement.^6^ As we-mentality is tested on three outcome variables, namely *attitudes*, *eng_local* and *eng_onl*, I correct for multiple hypothesis testing (MHT) using the conservative Holm-method (Holm, 1979). The adjusted p-value for the effect of we-menatlity on students’ local engagement is 0.03, indicating that the effect remains significant on the 5% level after this correction.

Using the eight survey items instead of the principal components in the multi-level regressions reveals a more detailed account of what is driving the results. Results are depicted in Appendix Tables 11, 12 and 13. It shows that we-mentality relates positively and significantly to the percentage of students being engaged in a local club or association (*mem_club*) as well as their willingness to take on a school office (*school*_*off*). The percentage of students taking on an honorary post outside of school (*hon_out_school*), however, is not related to the schools’ we-mentality.

With regard to the schools’ administrative dependency, students from private schools less strongly relate to the belief that it is important to make a change in society by being involved. In catholic schools, students are more often associated with taking on a school office or an honorary office outside of school. In schools that offer bilingual classes, more students are either engaged as a member of a club or association or take responsibility for an office in school (e.g. representative of the pupils). Finally, students attending rather large schools are more often engaged in a club or association. The same is true with engagement with an honorary office outside of school.

## Discussion

The paper’s main finding is that schools that express higher we-mentality exhibit more students that engage in civic activities within and outside of school. This result is robust to the choice of the model, i.e. standard OLS regressions or a multi-level approach that takes into account the nested structure of students within schools. As Barrett and Brunton-Smith (2014) argue: In order to understand the various factors (on different levels) influencing civic engagement, a multi-level approach is needed.

This is confirmed by the amount of variation of students’ civic attitudes and engagement that is explained by differences in schools. I found intraclass correlations (ICC) of between 3 and 8%. This is comparable to results from similar studies. Kahne and Sporte (2008) reported an ICC of 2.2%. While Quintelier (2010) reported a value of about 7%, Reichert and Print (2018) found values between 2.5% and 7.4% and Encina and Berger (2021), of around 11%. In this regard, the findings of this paper are consistent with previous research that schools explain a small but substantial part of students’ civic behavior.

A substantial amount of literature that investigates students’ well-being at school or their civic behavior focuses on schools’ social climate (among many others Castillo et al., 2015, Jagers et al., 2017, Schulz et al., 2017 and Encina & Berger, 2021). Guillaume et al. (2015) and Quin (2017) for instance measure teacher-student and student–student relationships directly from survey items and find a positive association between these relationships and students’ civic engagement. The concept and measurement of schools’ we-mentality used in this paper could be seen as a valuable complement to the study of schools’ social climate. If we-menatlity is promoted in the educational approach of a school, it is reasonable to theorize that this might also affect the school’s social climate.

The paper’s concept of schools’ we-mentality as their willingness and intention to promote we-ness and togetherness at school has also implications for the conceptualization of sense of community (Nowell & Boyd, 2010, 2014), sense of responsible togetherness (Procentese et al., 2019) and sense of belonging more general. The school context is special in this regard because students cannot only experience togetherness among themselves and with teachers but schools’ different educational approaches can more or less actively promote it. The result of this paper is consistent with Nowell and Boyd (2010) who stated “…when the community meets one’s needs, members will likely engage in a variety of important social outcomes such as becoming more civically involved…” (p. 834). From an educational science perspective, however, more research is needed to understand the exact mechanism in how schools can foster students’ civic behavior by their educational approach to we-mentality.

The study’s limitations can be summarized as follows. The survey was conducted with senior level students from secondary schools from two larger German cities. The results not necessarily generalize to younger students or to a more heterogenous sample, i.e. if schools from more rural areas would be added. An interesting avenue for future research would be to investigate geographical differences of the effect of we-mentality on students’ civic behavior as well as the age groups that are most responsive to a school’s approach in promoting togetherness.

Moreover, we-mentality is measured from a rather discursive form of statements that are published on the schools’ homepages. Taking the principles or a measure derived thereof as evidence for school officials’ or teachers’ actions or behavior has to be interpreted with caution. As stated earlier, it has to be assumed that those principles, teaching convictions and intention to promote we-ness are (at least partly) put into practice by school officials and teachers. In some cases, the schools substantiate their general principles by giving examples in how they promote we-ness through events at school, workshops, excursions and extracurricular activities with partners from outside of school (e.g. social projects). This could be seen as weak evidence for how the principles result into action. Not all schools of the sample, however, substantiate their principles with examples.

Finally, the study measured a school’s we-mentality but did not ask for students’ perception of we-ness at school. While measuring we-mentality from schools’ general principles offers an advantage for empirical identification, it would be interesting to investigate the interactions between schools’ promotion of we-ness and students’ perception thereof. These interactions, however, were beyond the scope of this study.

## Conclusion

This paper studies the effect of schools’ we-mentality on students’ civic attitudes and engagement. Civic attitudes and engagement are measured with eight items that stem from a survey conducted with 488 students in 13 German schools. We-mentality is defined as a school’s intention and willingness to promote a feeling of we-ness or togetherness among teachers and students as one of their guiding principles. Schools’ we-mentality is captured with a content-analysis approach that is applied to the general principle that is published on the schools’ homepage.

The main finding is that a school’s we-mentality is associated with students’ willingness to take on activities in their local community, i.e. being engaged in a local club or association or taking on a honorary post within or outside of their school. Moreover, students individual trust in others as well as willingness to try new things and attitudes towards party-democracy are related to their civic outcome. Overall, most of the variation in individual civic attitudes and engagement are associated with students’ individual characteristics such as trust in others, attitudes towards Europe and the party system and their willingness to engage with new and/or complicated tasks. School-level characteristics explain a rather small part of the overall variation.

The finding, however, that a school’s we-mentality is related to students’ local civic engagement within and outside of school, is intriguing and deserves scrutiny in further studies. If this result is confirmed by other studies, it would suggest enhancing civic education by improving the community-feeling and we-mentality at schools.

## Figures and Tables

**Fig. 2:**
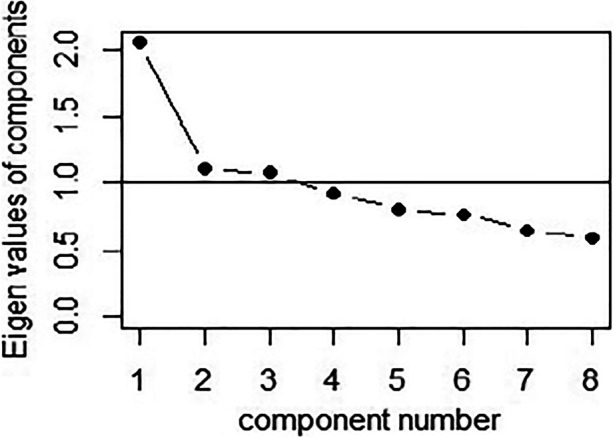
Scree plot

## Appendix 1

Table 7

**Table 7:** Measures of attitudes and engagement towards civic issues

No	Question	Variable name
	**Attitudes**	
1	Society is giving much to individuals I think, one should give something back. [7-Point likert scale: From totally agree to totally disagree]	soc_return
2	I think that it is important to be informed about what is happening in society and politics. [7-Point-likert scale: From totally agree to totally disagree]	informed_soc
3	I think that it is important to make changes happening in society by being involved. [7-Point likert scale: From totally agree to totally disagree]	change_soc
	**Engagement**	
4	Are you a member of an association or club? [Yes/No]	mem_club
5	Did you, during your time at school, apply being elected representative of the pupils of the school or applied for similar functions? [Yes/No]	school_off
6	Did you take a honorary post outside of school matters before? [Yes/No]	hon_out_school
7	Did you ever write a letter to the editor of a magazine/newspaper or formulated an article online with regard to a societal or political topic, e.g. you wrote a comment online on a (news) page? [Yes, frequently / Yes, once before / No]	write_let
8	How often do you have online discussions on (social) media with others on societal or political topics? [Very frequently, frequently, neither frequently nor infrequent, infrequent, very infrequent]	onl_media

The items were developed by the author to reflect civic attitudes and engagement that are suitable in the context of students

Table 8

**Table 8:** Measures of students’ individual attitudes

No	Question	Variable name
1	Imagine a ladder that represents the social hierarchy of the German society Where do you think your parents are located? [10-Point Likert scale from 1 to 10]	social ladder parents
2	Due to civil war in Syria, refugees flee to Germany. How strongly are your positive/negative attitudes towards refugees? [5-point Likert scale from strongly pronounced to not at all pronounced]	refugee attitudes
3	How likely will you spend time abroad within the next five years? [5-point Likert scale from very likely to very unlikely]	prob abroad
4	At the end of the survey, a lottery will decide that one of the participants wins an extra 10 Euros If you are chosen, how much would you donate to’Doctors without borders’? [Numerical value between 0 and 10]	donation
5	Do you think that most other people can be trusted or that you need be very careful with other people? [5-Point Likert sclale from you can trust most people to one needs to be very careful with other people]	trust others
6	How strongly do you agree with the following statement I like to engage with tasks if these are solvable. [5-Point Likert scale from totally agree to totally disagree]	solvable tasks
7	How strongly are your positive/negative attitudes towards party-democracy? [5-point Likert scale from strongly pronounced to not at all pronounced]	partydemo attitudes
8	How strongly are your positive/negative attitudes towards the European Union? [5-point Likert scale from strongly pronounced to not at all pronounced]	eu attitudes
9	How strong is your willingness to further engage with the topic of party-democracy topic? [5-point Likert scale from very high to very low]	partydemo willingness
10	How strong is your willingness to further engage with the topic of European Union topic? [5-point Likert scale from very high to very low]	eu willingness

The items were developed by the author

## Appendix 2

Results of the principal component analysis (PCA): The scree plot indicates three potential components, i.e. three eigenvalues of components are above one. A PCA with three components shows that individual civic attitudes load strongly on component one, local engagement measures on component two and online engagement measures on component three.

Figure 2

Table 9

**Table 9:** Results of PCA (*n* = 3 components)

Variables	Comp1	Comp2	Comp3	h2	u2	com
informed_soc	0.80			0.65	0.35	1.0
change_soc	0.66			0.55	0.45	1.5
soc_return	0.65			0.49	0.51	1.3
mem_club		0.69		0.51	0.49	1.1
hon_out_school		0.66		0.48	0.52	1.2
school_off		0.59		0.40	0.60	1.3
onl_media			0.78	0.61	0.39	1.0
write_let			0.71	0.56	0.44	1.2
SS loadings	1.55	1.40	1.31			
Proportion Var	0.19	0.17	0.16			
Cumulative Var	0.19	0.37	0.53			
Proportion Explained	0.36	0.33	0.31			
Cumulative Proportion	0.36	0.69	1.00			

The table displays the result of a principal component analysis (PCA). With regard to the factor loadings in the first part of the table, only loadings above 0.5 are displayed. The mean item complexity is 1.2

## Appendix 3

Table 10

**Table 10:** Dictionary used to detect “we-mentality”

German word	English translation
wir	we
uns	us
unser	our
zusammen	together
miteinander	with each other
füreinander	for each other
zusammenarbeit	collaboration
zusammenarbeiten	cooperate/work together
zusammenleben	living together
sozial	social
soziale	social
sozialen	social
soziales	social
gemeinwesen	community/collective
kooperativ	cooperative
kooperatives	cooperative
kooperative	cooperative
kooperieren	cooperate
kooperierend	cooperating
kooperierenden	cooperate
kooperiert	cooperate
kooperation	cooperation
kooperationsfähigkeit	abilty to cooperate
gemeinschaft	community/collective
schulgemeinschaft	school community
lerngemeinschaft	study group
gemeinsam	together
gemeinsamer	together
gemeinsamen	together
solidarität	solidarity
solidarisch	showing solidarity
hilfsbereit	helpful
hilfsbereitschaft	helpfulness
mitwirken	collaborate
kollegial	cooperative/loyal
kollegiale	cooperative/loyal
emphatie	empathy
empathiefähigkeit	ability for empathy
mitmenschen	fellow men
teamfähigkeit	ability to work in a team
teamgeist	team spirit
zusammenwachsen	coalescence
gegenseitig	mutual
gegenseitige	mutual
gegenseitigem	mutual
nächstenliebe	altruism/charity
zwischenmenschlich	interpersonal
zusammengehörigkeitsgefühl	feeling of belonging together

The dictionary was developed by the author

## Appendix 4

Table 11

**Table 11:** Multi-level with attitude variables

	soc_return	informed_soc	change_soc
female	− 0.053	0.028	0.152^∗∗^
	(0.088)	(0.054)	(0.068)
age	− 0.023	− 0.061^∗∗^	0.058
	(0.049)	(0.030)	(0.038)
social ladder parents	− 0.002	0.021	− 0.084^∗^
	(0.059)	(0.037)	(0.046)
refugee attitudes	0.013	0.002	0.080^∗∗∗^
	(0.026)	(0.016)	(0.021)
prob abroad	0.030	0.009	0.057^∗∗^
	(0.033)	(0.021)	(0.026)
donation	0.025^∗∗^	− 0.008	0.008
	(0.011)	(0.007)	(0.008)
trust others	0.173^∗∗∗^	0.024	0.042
	(0.040)	(0.025)	(0.031)
try new things	0.082^∗^	0.066^∗∗^	0.076^∗∗^
	(0.047)	(0.029)	(0.037)
solvable tasks	0.055	− 0.006	0.010
	(0.041)	(0.026)	(0.032)
partydemo attitudes	0.066^∗∗∗^	0.039^∗∗^	0.005
	(0.025)	(0.016)	(0.020)
eu attitudes	0.018	0.023	− 0.013
	(0.027)	(0.017)	(0.021)
partydemo willingness	0.077^∗^	0.120^∗∗∗^	0.157^∗∗∗^
	(0.046)	(0.028)	(0.036)
eu willingness	0.128^∗∗∗^	0.067^∗∗^	− 0.012
	(0.049)	(0.030)	(0.038)
size	0.0001	− 0.0001	− 0.0002
	(0.0002)	(0.0001)	(0.0002)
we-mentality	− 0.112	0.257	0.215
	(0.305)	(0.179)	(0.227)
private	0.007	− 0.158^∗^	− 0.214^∗∗^
	(0.148)	(0.085)	(0.108)
catholic	− 0.119	0.086	0.222^∗^
	(0.173)	(0.102)	(0.129)
music	0.004	0.052	− 0.134
	(0.201)	(0.118)	(0.150)
naturalscience	0.137	− 0.139	0.053
	(0.175)	(0.102)	(0.129)
bilingual	0.050	0.030	0.097
	(0.159)	(0.093)	(0.118)
constant	1.126	4.209^∗∗∗^	1.560^∗∗^
	(1.011)	(0.619)	(0.785)
Obs	486	486	486
Groups (school id)	13	13	13
ICC	0.00	0.00	0.00
Log Likelihood	− 618.632	− 383.824	− 499.289
AIC	1,283.263	813.648	1,044.579
BIC	1,379.546	909.931	1,140.861

The table reports results of multi-level regressions with *soc return*, *informed soc* and *change* *soc* as the dependent variables. Explanatory variables are on the individual- and school-level. Regression coefficients are presented together with standard errors reported in parentheses. * indicates significance at the 10% level, ** at the 5% level and *** at the 1% level

Table 12

**Table 12:** Multi-level with local engagement variables

	mem_club	school_off	hon_out_office
female	− 0.004	− 0.023	− 0.001
	(0.048)	(0.048)	(0.048)
age	0.022	− 0.002	0.065^∗∗^
	(0.027)	(0.027)	(0.027)
social ladder parents	0.029	0.026	0.041
	(0.032)	(0.032)	(0.032)
refugee attitudes	− 0.024^∗^	− 0.018	− 0.005
	(0.014)	(0.014)	(0.014)
prob abroad	0.017	0.041^∗∗^	0.035^∗^
	(0.018)	(0.018)	(0.018)
donation	0.001	0.004	0.007
	(0.006)	(0.006)	(0.006)
trust others	0.022	0.017	− 0.014
	(0.022)	(0.022)	(0.022)
try new things	− 0.005	0.013	0.025
	(0.026)	(0.026)	(0.026)
solvable tasks	0.011	0.045^∗∗^	0.070^∗∗∗^
	(0.023)	(0.023)	(0.023)
partydemo attitudes	0.016	0.020	− 0.010
	(0.014)	(0.014)	(0.014)
eu attitudes	− 0.014	0.022	0.017
	(0.015)	(0.015)	(0.015)
partydemo willingness	0.027	0.048^∗^	0.051^∗∗^
	(0.025)	(0.025)	(0.025)
eu willingness	0.039	− 0.011	− 0.004
	(0.027)	(0.027)	(0.027)
size	0.0005^∗∗∗^	− 0.00004	0.0002^∗∗^
	(0.0001)	(0.0001)	(0.0001)
we-mentality	0.344^∗∗^	0.425^∗∗∗^	0.195
	(0.158)	(0.157)	(0.157)
private	0.161^∗∗^	− 0.047	− 0.028
	(0.075)	(0.075)	(0.075)
catholic	− 0.077	0.238^∗∗∗^	0.218^∗∗^
	(0.090)	(0.090)	(0.090)
music	0.097	0.129	0.086
	(0.104)	(0.104)	(0.104)
naturalscience	− 0.053	− 0.163^∗^	− 0.084
	(0.090)	(0.090)	(0.090)
bilingual	0.171^∗∗^	0.194^∗∗^	0.131
	(0.082)	(0.082)	(0.082)
constant	− 0.813	− 0.407	− 1.719^∗∗∗^
	(0.548)	(0.546)	(0.546)
Obs	485	486	486
Groups (school id)	13	13	13
ICC	0.00	0.00	0.00
Log Likelihood	− 322.797	− 322.389	− 322.627
AIC	691.595	690.778	691.255
BIC	787.830	787.061	787.537

The table reports results of multi-level regressions with *mem*_*club*, *school_off* and *hon_out_school* as the dependent variables. Explanatory variables are on the individual- and school-level. Regression coefficients are presented together with standard errors reported in parentheses. * indicates significance at the 10% level, ** at the 5% level and *** at the 1% level

Table 13

**Table 13:** Multi-level with online engagement variables

	write_let	onl_media
female	− 0.101^∗∗^	− 0.148
	(0.048)	(0.117)
age	− 0.027	− 0.004
	(0.027)	(0.066)
social ladder parents	0.032	− 0.006
	(0.033)	(0.079)
refugee attitudes	0.025^∗^	0.022
	(0.015)	(0.035)
prob abroad	0.050^∗∗∗^	0.011
	(0.018)	(0.044)
donation	− 0.0002	0.010
	(0.006)	(0.014)
trust others	0.024	0.011
	(0.022)	(0.054)
try new things	0.026	0.066
	(0.026)	(0.063)
solvable tasks	− 0.013	0.036
	(0.023)	(0.055)
partydemo attitudes	− 0.004	− 0.009
	(0.014)	(0.034)
eu attitudes	0.012	− 0.040
	(0.015)	(0.036)
partydemo willingness	0.028	0.270^∗∗∗^
	(0.025)	(0.061)
eu willingness	0.016	0.052
	(0.027)	(0.065)
size	− 0.0003^∗∗^	− 0.001^∗∗∗^
	(0.0001)	(0.0003)
we-mentality	0.143	0.179
	(0.159)	(0.386)
private	− 0.077	− 0.226
	(0.076)	(0.184)
catholic	0.166^∗^	0.282
	(0.091)	(0.220)
music	− 0.160	− 0.688^∗∗∗^
	(0.105)	(0.255)
naturalscience	− 0.025	0.399^∗^
	(0.091)	(0.221)
bilingual	0.111	− 0.058
	(0.083)	(0.201)
constant	0.381	1.925
	(0.552)	(1.337)
Obs	486	486
Groups (school id)	13	13
ICC	0.00	0.00
Log Likelihood	− 328.426	− 758.208
AIC	702.852	1,562.416
BIC	799.135	1,658.699

The table reports results of multi-level regressions with *write*_*let* and *onl_media* as the dependent variables. Explanatory variables are on the individual- and school-level. Regression coefficients are presented together with standard errors reported in parentheses. * indicates significance at the 10% level, ** at the 5% level and *** at the 1% level

## Footnotes

1 A good example is the international climate movement Fridays for Future.

2 This usually also means that the school is (at least partly) funded by the catholic church.

3 As an alternative, one could have thought of using the textual content from the entire school’s website. Beside the general principle, however, the websites are very different in structure and focus. This would potentially add noise to the measure that is calculated with the general principles.

4 Results from OLS regressions using the eight survey items instead of principal components detail which specific item drive the results with regard to effects of individual characteristics in Table 4. Results are available upon request.

5 One could also think of the class environment as the second level that exhibits an influence on students’ civic attitudes and engagement. 11th and 12th graders from German schools taking part in the study, however, do not attend fixed classes anymore such as lower graders. They rather attend courses that vary in the composition with regard to students and teachers. Therefore, I abstain from using the class as the second level but rather the school.

6 Results are robust using simple OLS regressions with student- and school-level characteristics but without fixed effects. Moreover, the results are also robust using a more simplistic measure of we-mentality by only considering the words ”we”, ”us” and ”our” from Table 10. Results of both robustness checks are available upon request.
